# Removal of Interictal MEG-Derived Network Hubs Is Associated With Postoperative Seizure Freedom

**DOI:** 10.3389/fneur.2020.563847

**Published:** 2020-09-24

**Authors:** Sriharsha Ramaraju, Yujiang Wang, Nishant Sinha, Andrew W. McEvoy, Anna Miserocchi, Jane de Tisi, John S. Duncan, Fergus Rugg-Gunn, Peter N. Taylor

**Affiliations:** ^1^Interdisciplinary Computing and Complex BioSystems Group, CNNP Lab, School of Computing, Newcastle University, Newcastle upon Tyne, United Kingdom; ^2^Department of Clinical and Experimental Epilepsy, UCL Queen Square Institute of Neurology, London, United Kingdom; ^3^Faculty of Medical Science, Newcastle University, Newcastle upon Tyne, United Kingdom

**Keywords:** epilepsy, surgery, network, MEG (magnetoencephalography), outcome prediction

## Abstract

**Objective:** To investigate whether MEG network connectivity was associated with epilepsy duration, to identify functional brain network hubs in patients with refractory focal epilepsy, and assess if their surgical removal was associated with post-operative seizure freedom.

**Methods:** We studied 31 patients with drug refractory focal epilepsy who underwent resting state magnetoencephalography (MEG), and structural magnetic resonance imaging (MRI) as part of pre-surgical evaluation. Using the structural MRI, we generated 114 cortical regions of interest, performed surface reconstruction and MEG source localization. Representative source localized signals for each region were correlated with each other to generate a functional brain network. We repeated this procedure across three randomly chosen one-minute epochs. Network hubs were defined as those with the highest intra-hemispheric mean correlations. Post-operative MRI identified regions that were surgically removed.

**Results:** Greater mean MEG network connectivity was associated with a longer duration of epilepsy. Patients who were seizure free after surgery had more hubs surgically removed than patients who were not seizure free (AUC = 0.76, *p* = 0.01) consistently across three randomly chosen time segments.

**Conclusion:** Our results support a growing literature implicating network hub involvement in focal epilepsy, the removal of which by surgery is associated with greater chance of post-operative seizure freedom.

## Introduction

Epilepsy affects 50 million people worldwide, with one third not responding to medication. Neurosurgical treatment is potentially curative in focal epilepsy, if the source of the epilepsy, “the epileptogenic zone” can be identified and removed. The goal of pre-surgical evaluation is to identify the epileptogenic zone using semiology, neuroimaging, and neurophysiology (1). However, despite the abundant data that are used to inform clinical decision making, around 50–70% of patients continue to experience post-operative seizures. The possibility of postoperative seizures, and risks of adverse effects, acts as a significant barrier to surgery as some patients who may benefit do not proceed to operation. The ability to better predict if patients will have favorable post-operative outcomes (in terms of seizure-freedom) would therefore be highly beneficial.

The identification of a measure for accurate outcome prediction is challenging, in part due to the complexity of brain network interactions. Functional brain networks derived from magnetoencephalography (MEG) data can be inferred by computing the pairwise similarity of brain regions. Several studies have shown increased MEG functional connectivity in patients with epilepsy compared to controls, even in inter-ictal periods (2–6). In two separate studies, Jin et al. (7) showed altered MEG network “hubs” —those regions with high network connectivity—in temporal areas in patients with hippocampal sclerosis, and increased network efficiency in patients with focal cortical dysplasia (8). With respect to surgical outcomes, Nissen et al. (9) investigated if MEG network hubs overlapped more with the resection area in seizure-free patients. The authors reported that hubs were localized within the area later resected in 9 of 14 seizure free patients, but none of the patients who had post-operative seizures. A later study from the same group showed in a larger cohort of 94 patients that areas with increased functional connectivity significantly overlapped with tissue that was later resected, but was not associated with outcomes. The study by Englot et al. (10) also demonstrated increased correlations in areas that were later resected in patients with good outcomes. Aydin et al. (11) suggested MEG networks could be used to predict outcome, and Krishan et al. (12) suggested epileptogenic source localization is feasible using MEG connectivity analysis irrespective of the presence/absence of inter-ictal spikes.

In addition to surgical outcomes, MEG network properties have been related to epilepsy duration (i.e., number of years a patient has had epilepsy), or age of onset. For example, Englot et al. (10) showed overall network connectivity to be negatively associated with epilepsy duration whilst in contrast Madhavan et al. (13) showed a positive association. Jin et al. (8) showed a negative association with age of seizure onset.

Taken together the MEG network literature suggests an increased connectivity in patients, particularly in “hub” areas that are later resected, which may be related to outcome and associated with duration. In this study we investigate MEG hubs and their removal in a cohort of 31 patients. Furthermore, since within-patient consistency is a critical step required before clinical application, we assessed the consistency of these results across three different time segments across four different parcellations. We hypothesized that the removal of high strength nodes would result in seizure-freedom. Our findings support earlier literature of being strong involvement of hub nodes in epileptogenic networks (14).

## Methods

### Patients

We retrospectively analyzed data from 31 patients who underwent pre-operative evaluation and subsequent epilepsy surgery at the National Hospital for Neurology and Neurosurgery, Queen Square, London. Outcomes of seizure freedom were assessed at least 12 months post-operatively according to the ILAE classification (15). In this cohort, 19 of the 31 patients had post-operative seizures (ILAE 2 or greater). All patients had no prior neurosurgery. There was no significant difference between outcome groups in terms of age, sex, or location of resection (Mann-Whitney *U*-test for age, χ^2^-test for differences in location, side, and sex) (see Table 1 for a summary and Supplementary Table 1 for detailed patient information).

**Table 1:** Patient demographics and relation to outcome group.

	**ILAE 1**	**ILAE 2-5**	**Significance**
*N* (%)	12 (39%)	19 (61%)	
Temporal/extratemporal	9/3	10/9	χ^2^ = 2.3, p=0.13
Left/right hemisphere	8/4	11/8	χ^2^ = 1.8, *p* = 0.18
Age (mean, SD)	34.3, 10.5	31.3, 10	*P* = 0.37
Sex (M/F)	6/6	13/6	χ^2^ = 1.1, *p* = 0.3
Recording length in minutes (mean,SD)	15, 6.04	17.78, 7.12	*P* = 0.17

### MRI Acquisition and Processing

A T1 weighted MRI was acquired for all patients pre- and within 12 months post-operatively using a 3T GE Signa HDx scanner (General Electric, Waukesha, Milwaukee, WI). Standard imaging gradients with a maximum strength of 40 mTm^−1^ and slew rate 150 Tm^−1^ s^−1^ were used. All data were acquired using a body coil for transmission, and 8- channel phased array coil for reception. The high resolution T1-weighted volumetric image was acquired with 170 contiguous 1.1 mm-thick slices (matrix, 256 × 256; in-plane resolution, 0.9375 × 0.9375 mm).

The preoperative MRI was used to generate cortical regions using the standard FreeSurfer recon-all pipeline (16). In brief, this performs intensity normalization, skull stripping, subcortical volume generation, gray/white segmentation, and parcellation (16–18). Surfaces were visually inspected and manually corrected where necessary. We then generated labels for the cortex from the Lausanne multi-resolution parcellation (https://github.com/mattcieslak/easy_lausanne. (19) using surface-based registration. This resulted in four different parcellations which were later used to generate four networks per subject. These parcellations have different numbers of regions. The most coarse contains 68 regions and is the Desikan-Kiliany parcellation, which is based on anatomical boundaries (18), and the finest containing 448 regions which are subdivisions of lower resolution parcellations.

To identify which regions were affected, and which were completely spared by the resection we linearly registered the post-operative MRI to the pre-operative MRI using the FSL FLIRT tool (6 degrees of freedom) (20, 21). We then overlaid the post-operative MRI on the pre-operative MRI using FSLview and manually drew a mask to delineate which tissue was resected (22). Care was taken to ensure masks were not extended beyond, or reduced in proximity to, boundaries known to be resected (e.g., beyond the sylvian fissure for a temporal lobe resection). Care was also taken to account for any brain shift as in our previous study (22). For one patient (IDP 851), a post-operative MRI was unavailable and the surgery report from the clinical team was therefore used to identify the resected tissue mask. The masks were then overlaid on the volumetric regions of interest from the Lausanne parcellation listed above. Regions overlapping the mask were labeled as removed, and others labeled as spared by surgery. If any overlap was present, then the region was considered as removed.

### MEG Acquisition and Processing

MEG data for all experiments were recorded using a 275-channel CTF Omega whole head gradiometer system (VSM MedTech) in a magnetically shielded environment, with a 600-Hz sampling rate. After participants were comfortably seated in the MEG, head localizer coils were attached to the nasion and preauricular regions, 1 cm anterior to the left and right tragus to monitor head movement during the recording sessions. Patients were requested to keep as still as possible during the individual 6-min recording epochs, awake and their eyes closed in resting state. No patient had any overt seizure during the MEG recordings. The total length of MEG recording differed between patients (mean 16.70 min, standard deviation 6.82 min). Although the MEG recording duration was slightly different for each patient, this did not differ significantly between the outcome groups (Table 1).

**Figure 1:**
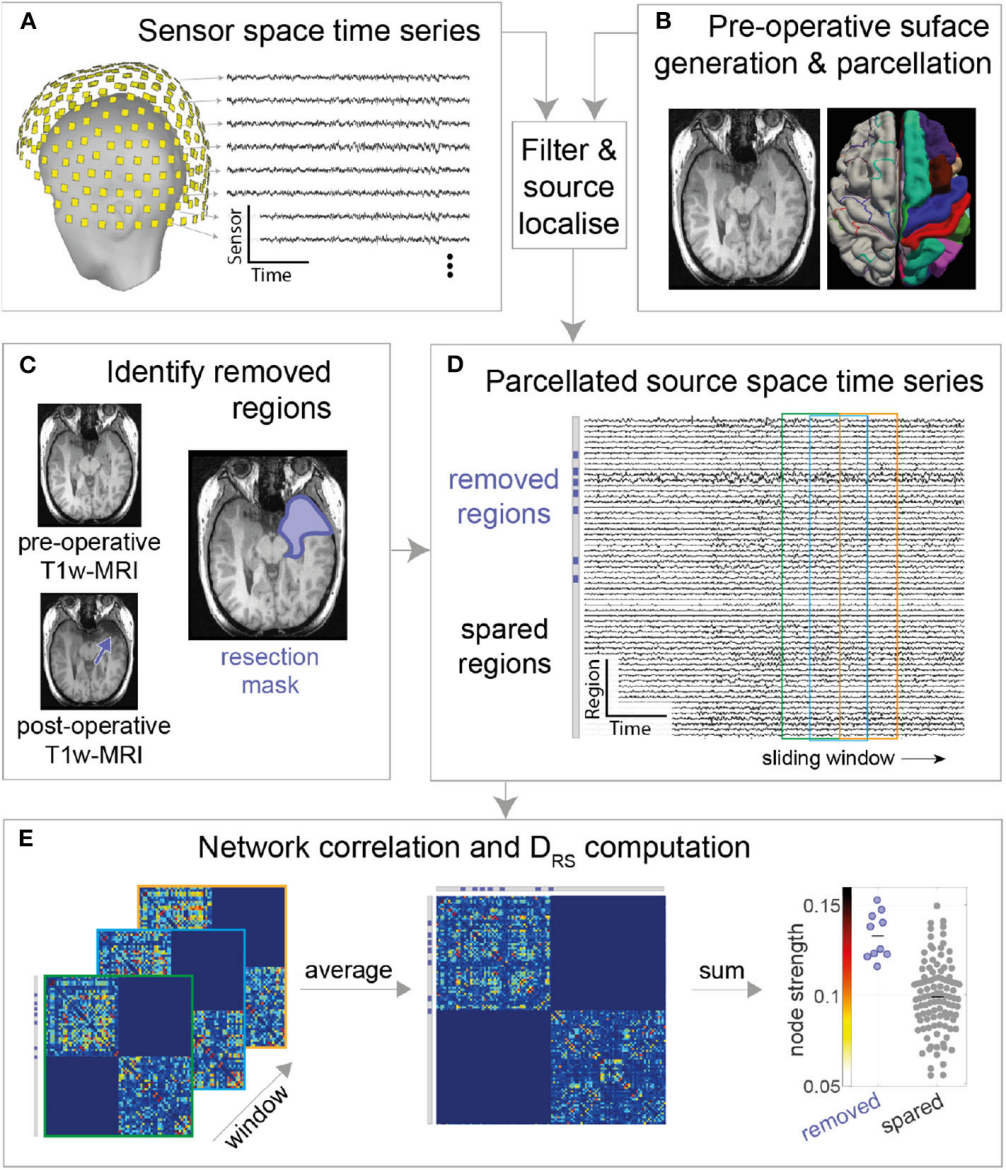
MEG processing pipeline. **(A)** Resting state MEG sensor data is filtered and **(B)** source localized using pre-operative MRI followed by parcellation **(C)** Post-operative T1-weighted MRI is overlaid on pre-operative T1-weighted MRI to obtain resection mask **(D)** Each parcellated source time series is now labeled as removed or spared using resection mask. A functional connectivity matrix is obtained for every sliding window (2s window with 50% overlap) **(E)** Functional connectivity matrices are then averaged to obtain a single connectivity matrix. Node strength is obtained from the averaged connectivity matrix, followed by D_RS_ calculation between Spared and Removed regions.

The MEG sensor data were pre-processed in two steps using the Brainstorm software (23). First, data were notch filtered at 50 Hz (24) (IIR 2nd order zero phase-lag, Brainstorm implementation) followed by bandpass filtering (zero phase-lag even-order linear phase FIR filter, based on a Kaiser window design, Brainstorm implementation) the data between 1 and 55 Hz (broadband) using Brainstorm's pre-processing module (Figure 1A). Second, the band-passed data were decomposed into different components using ICA, followed by removal of eye blink and cardiac components. The co-registration MEG helmet with pre-operative T1w MRI scan was performed using fiducials (anion, nasion, and preauricular points).

MEG data were source reconstructed using sLORETA, a distributed model with zero localization error (25). The forward model (head model) was built using an overlapping multiple local sphere head model, which has accuracy similar to a boundary element model but is orders of magnitude faster to compute (10, 26), with 15,000 voxels constrained perpendicular to the cortical surface. These 15,000 voxels are divided into cortical regions of interest (ROIs) using the Laussane parcellation schemes. We derived one time series per ROI using a flipped mean approach (23), resulting in, for the scale 114 parcellation, a 114 x number of time points matrix (Figure 1D). The pre-processing pipeline is visually explained in Figure 1.

### Network Construction and Analysis

Three one-minute epochs (10), each separated by at least five minutes, were chosen randomly for each patient. Given that some patients had insufficient durations of artifact-free recording our sample size reduced for epochs two and three. The results presented in the main text are for the first epoch, with others shown in Supplementary Materials. A two-second sliding window with 50% overlap was computed over the source reconstructed time series, extracting one functional connectivity (amplitude correlation using Pearson correlation) matrix per 2-s window (Figure 1E; left panel). The time varying functional connectivity matrices were temporally averaged across windows to one matrix, which represents the functional network of the entire epoch. The same was repeated for each of the other two epochs. Figures 1D,E summarizes the methods. The intra-hemispheric node strength, which we defined as the mean correlation of the nodes within hemisphere, was calculated. Nodes with high node strengths (large positive values) were hypothesized to be epileptogenic and thus their subsequent removal hypothesized to result in better patient outcomes.

As all patients had unilateral resections i.e., resection in only one hemisphere, we posited that connectivity ipsilateral to the epileptogenic zone is stronger than connectivity within the contralateral hemisphere. We therefore excluded inter-hemispheric connectivity (Figure 1E) since we expected this to hold less discriminatory information. We note that field spread may lead to spurious correlations; however, since the same methods are applied to all patients we do not expect this to be a confound to explain either outcome or duration.

**Figure 2:**
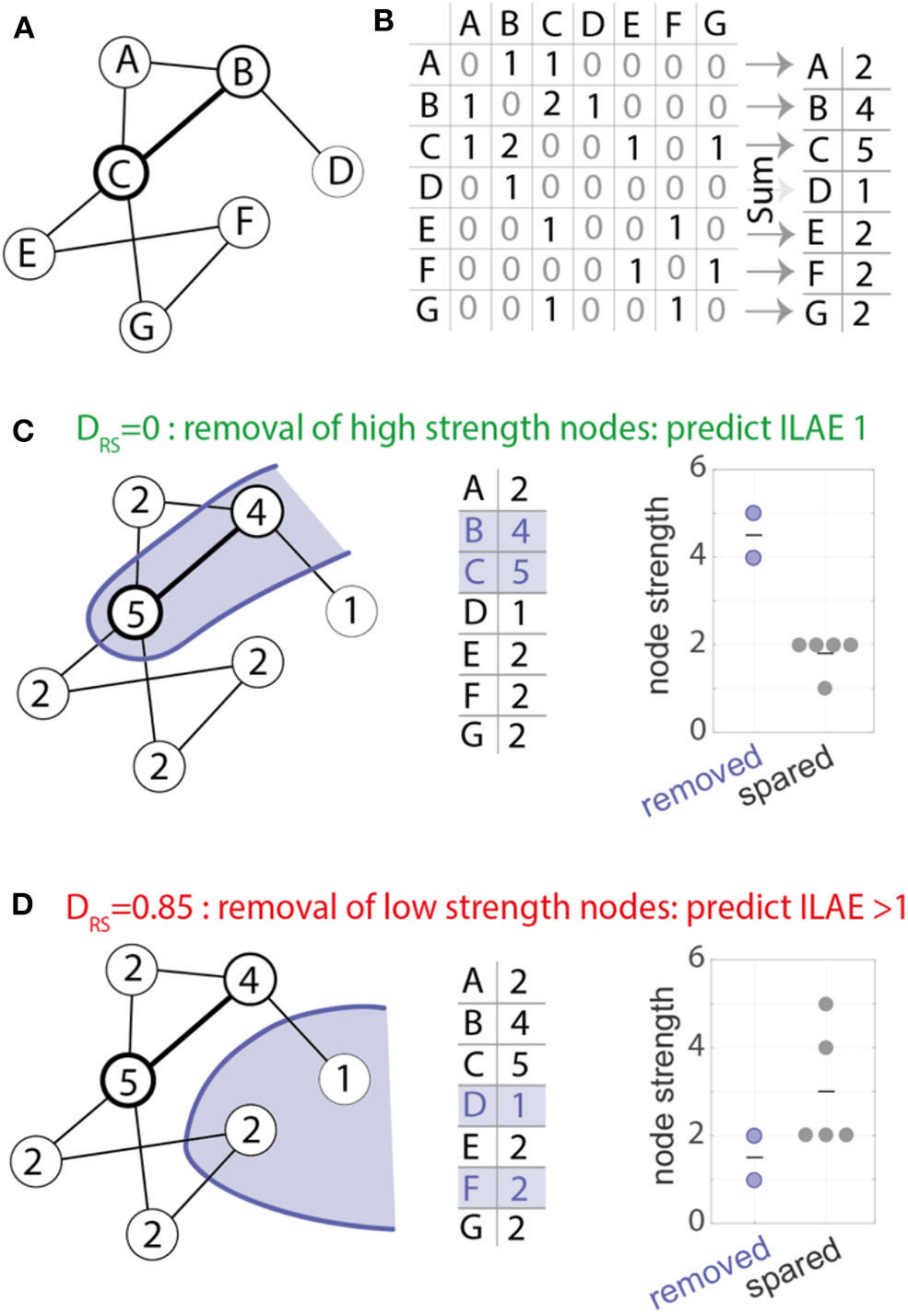
Computation of the D_RS_ measure. **(A)** example network used for demonstration with seven nodes and eight weighted bidirectional connections. **(B)** The network in **(A)** represented as a weighted connectivity matrix. Taking the sum across the rows of the matrix gives a node strength value for each node. **(C)** The nodes in the network show the node strength. For example, node A has strength 2, node B has strength 4 and so on. An example resection to nodes B and C (shown in blue) would remove the two most strongly connected nodes (respective node strength 4 and 5, middle panel). Removed and spared nodes can be perfectly distinguished from each other—all removed nodes have higher node strength than all spared nodes. **(D)** Alternative resection scenario where the removal of low strength nodes leads to a D_RS_ value of 0.85. In the scenario where removed and spared nodes have identical strength values a D_RS_ value of 0.5 would be measured.

To quantify the difference in node strengths between removed and spared tissue we used the area under receiver operator statistic curve (AUC) which is equivalent to the normalized non-parametric Mann-Whitney *U* statistic, we term this measure as D_RS_ (Distinguishability of Removed node strength vs. Spared node strength). A D_RS_ value of 0 or 1 indicates complete distinguishability of resected tissue from spared tissue. A value of 0 indicates that the strength of all resected nodes is higher than all spared nodes. In contrast, a value of 1 indicates that the strength of all spared nodes is higher than all resected nodes. Any value around 0.5 indicates similar rank-ordering of node strengths from both tissue types (27). This is a non-parametric method, which is robust to outliers and is effective even with non-gaussian distributions and generates a single value of D_RS_ per patient (Figure 2 provides an illustrative example). This measure was introduced by Wang et al. (27).

### Statistical Analysis

The area under the receiver operating curve (AUC) measure was used to determine whether D_RS_ distinguishes seizure-free (ILAE 1) from not-seizure-free (ILAE>1) outcomes. If the AUC = 1, or if AUC = 0, then there is the highest separation between good and bad outcomes, on the contrary, if AUC = 0.5, then separation is by chance. Hypothesizing that ILAE 1 patients would have lower D_RS_ values (see Figure 2) (10, 27), we used a one-tailed Mann-Whitney *U*-test (ranksum) test to compare the difference between outcome groups. We computed 95% confidence intervals of the AUC using a logit transformation (28).

To test associations between the mean of the entire functional connectivity matrix i.e., average of all the connections (mean-FC) and log_10_ epilepsy duration (DUR), we compared the following two regression models under the likelihood ratio test to obtain a *p*-value for each time segment:

Model: mean FC ~ 1+ DUR vs Model_0_: mean FC ~ 1.

This tests if log_10_ epilepsy duration explains any of the variance in FC across subjects. The regression models were fitted using a robust regression approach.

To test if duration and mean FC were positively associated over all segments, we used a linear mixed effects model, where we modeled the time segment as a random effect:

LME: mean FC ~ 1+ DUR + (1|segment).

To test if duration explained significantly more variance in mean FC, we tested it against the alternative model LME_0_: mean FC ~ 1 + (1|segment) using a likelihood ratio test again.

## Results

### High Connectivity in Resected Tissue Is Associated With Favorable Outcome

**Figure 3:**
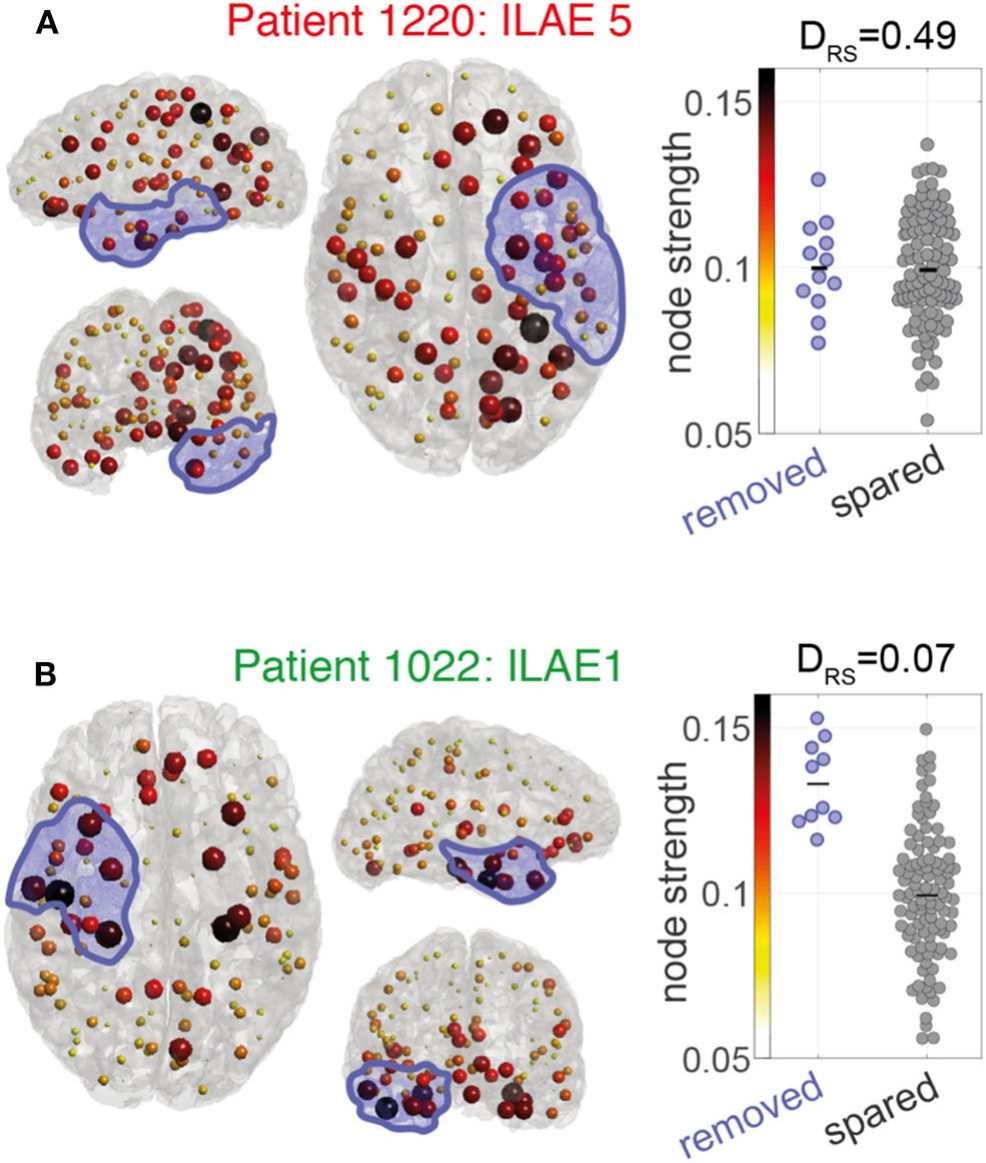
Node strength visualization for two patients **(A,B)**. Left and center panels: Node strength is visualized as the color and size of each ROI marker. Larger (darker) spheres indicate ROIs with higher MEG interictal network node strength. Blue shaded area indicates the surgically removed tissue. Right panels: Beeswarm plot of the MEG interictal network node strength in removed (blue) vs. spared (gray) ROIs. Each data point is the node strength of an individual region. The D_RS_ value is a measure of effect size to indicate differences in the node strengths of removed and spared nodes. A D_RS_ of 0 indicates that the removed ROIs all have larger node strengths than the spared ROIs. A D_RS_ of around 0.5 means that both ROI types have similar levels of node strength.

We hypothesized that the removal of high strength nodes would result in seizure-freedom. Figure 3 shows the pre-operative node strength for two example patients and their later resections overlaid in blue. Patient 1220 (Figure 3A) had a left sided temporal lobe resection, with many non-resected high-strength nodes in parietal and frontal areas. The distinguishability between the resected and spared node strengths (lower panel) is close to 0.5 which suggests the removed nodes are no different to the rest of the network in their strength. This patient had post-operative seizures with an ILAE outcome class 5.

In contrast, patient 1022 (Figure 3B) had a right sided temporal lobe resection and was seizure-free afterwards. Many of the highest strength “hub” nodes lay within the resection zone meaning they were subsequently removed by surgery. A D_RS_ sore of 0.07 reflects that (i) removed nodes tend to have higher strength than spared nodes and (ii) the difference between removed and spared node is large.

### Consistency of Findings

**Figure 4:**
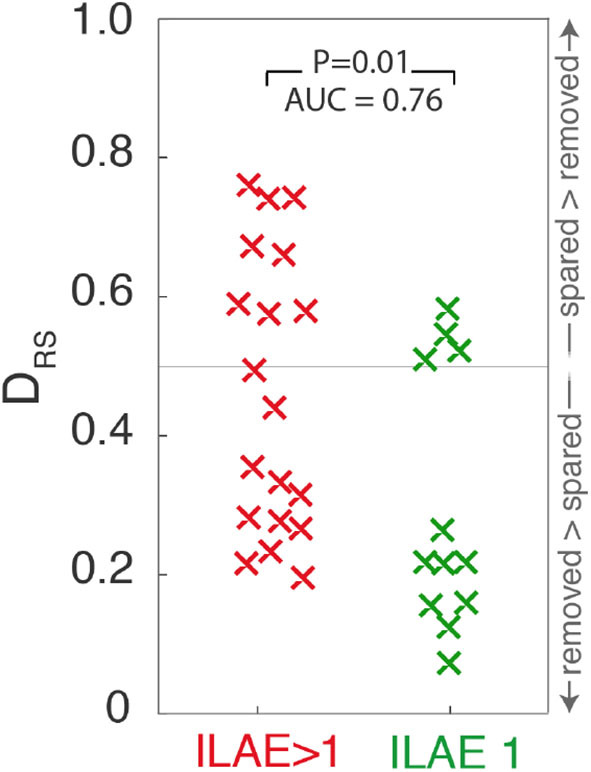
Scatter plot depicting D_RS_ values for ILAE1 and ILAE>1 surgical outcomes. Values close to 0 (1) indicate that high strength nodes are resected (spared). Each “x” marker represents an individual patient.

Extending the analysis to include all 31 patients shows significantly lower D_RS_ values (i.e., the resected and spared ROIs are more distinguishable) in patients with post-operative seizure-freedom, compared to those who were not seizure-free (one tailed Wilcoxon rank sum *p* = 0.01, AUC = 0.76, 95%CI = 0.54–0.90, Figure 4). Confusion matrices for the ROC curve optimized for maximum accuracy are shown In Supplementary Table 2)

To test the temporal robustness of this result the entire analysis was repeated for two additional one-minute segments, separated by at least five minutes. Supplementary Table 2 shows these findings for the 28 patients for whom there were sufficient data available to repeat the analysis. For both of those additional segments, ILAE 1 patients still had significantly lower D_RS_ values than those patients with ILAE>1 outcomes (segment 2: *p* = 0.04, AUC = 0.7, 95%CI = 0.47–0.86, segment 3: *p* = 0.02, AUC = 0.74, 95%CI = 0.51–0.88).

The networks generated in Figure 4 comprise cortical networks based on 114 ROIs using the Lausanne subparcellation of the original Desikan-Kiliany (DK) atlas (18). Repeating the analysis for higher resolution subparcellations, comprising 219, or 448 regions, the finding of lower D_RS_ values for ILAE1 patients is replicated for this time segment (*p* < 0.05, Supplementary Table 1), but was not significant for other time segments, or when using the DK parcellation (Supplementary Table 3).

### Higher Connectivity Is Associated With Longer Duration

**Figure 5:**
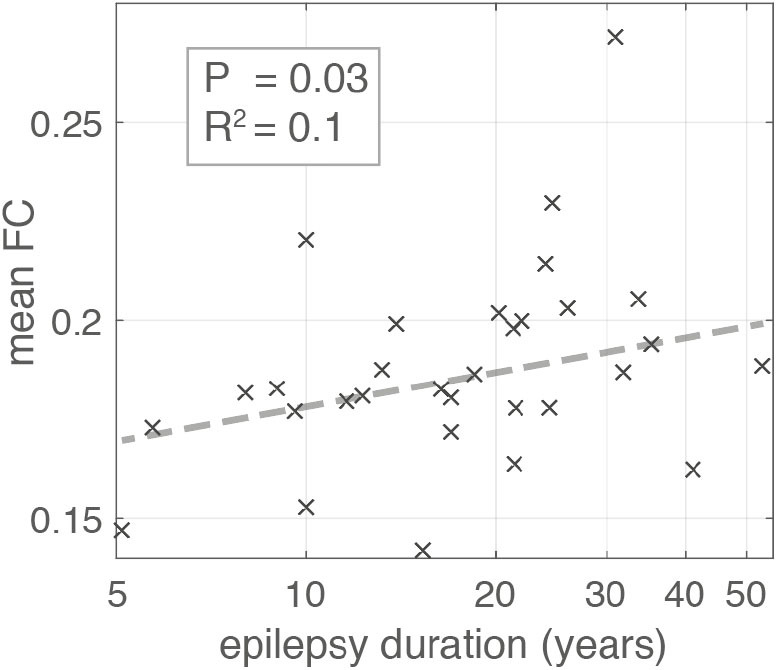
Scatter plot illustrating the relationship between Epilepsy duration in years and mean global functional connectivity. Each “x” marker represents an individual patient. Dashed line represents the line of best fit using bisquare linear regression robust to outliers. The association is significant with *p*-value = 0.03 (likelihood ratio test) and adjusted *R*^2^ = 0.1.

Using a linear regression model robust to outliers we found the duration of epilepsy was positively associated with mean FC in contrast to the negative association reported by Englot et al. (10) (Figure 5) (likelihood ratio test *p* = 0.03, adjusted *R*^2^ = 0.1). The positive association was also present for time segment 3 (likelihood ratio test *p* ≪ 0.01, adjusted-*R*^2^ = 0.17), but was not significant for segment 2 (likelihood ratio test *p* = 0.1, adjusted *R*^2^ < 0.01). Given the conflicting result from segment 2 we applied a linear mixed effects model that incorporates the segment number as a random effect in the analysis, boosting overall statistical power utilizing all available data. This approach found a significant positive association overall between duration and mean FC (likelihood ratio test *p* = 0.02).

## Discussion

In this study we investigated pre-operative functional connectivity networks, constructed in source space, using MEG recordings from 31 patients with refractory focal epilepsy who later underwent epilepsy surgery. Networks were constrained by pre- and post-operative MRI allowing accurate delineation of resected regions. We report three main findings. First, seizure-free patients showed higher preoperative node strength in surgically-removed regions compared to surgically-spared regions. Second, capturing this discrepancy between surgically-removed and surgically-spared regions patient-specifically by our proposed D_RS_ measure, we found significant differences in D_RS_ between outcome groups. Third, overall network connectivity strength showed a weak, but significant, positive association with epilepsy duration.

Our approach builds on our previous work with intracranial EEG, where we show that patients generally have better seizure outcomes when high-strength nodes are surgically removed (27–30). Other groups have reported similar findings from intracranial EEG (31–37). With MEG data, Nissen et al. (9) showed in a cohort of 22 patients, that interictal source localized network hubs overlapped with the resection in seizure-free patients only. There, the authors applied node betweenness centrality (38) as their measure of hubness, as opposed to our measure of node strength. Node strength and betweenness centrality are highly correlated, so our results are in strong agreement. Jin et al. (39) used similar methods (nodal betweenness centrality) applied to source localized interictal MEG and reported altered network hubs in patients, as compared to healthy controls. Englot et al. (10) reported increased connectivity in the resected region to be more frequent in seizure-free patients. Our findings support a strong involvement of hub nodes in epileptogenic networks (14).

We investigated the robustness of our results to the choice of time segment. This is important clinically because it is unknown if there is an optimal time segment, or whether results may vary over time. Our finding of consistent differences in D_RS_ values between outcome groups, regardless of time segment suggests confidence that segments of one-minute duration are sufficient. Other previous studies have also found one minute to be sufficient for consistent predictions in most cases (10, 27), and that short durations are sufficient to capture stationary aspects of the functional connectivity (40).

In contrast to the consistency across time segments, we found some variability associated with the choice of spatial parcellations. Although a trend of increased D_RS_ in poor outcome patients was present in all analyses (Supplementary Table 1), this only consistently met significance across all segments for only the 114 ROI parcellation. This may reflect a compromise between the regions being small enough to not have averaging across large regions (incurring a loss of data), but still large enough to represent independent time series data for our cohort as a whole. However, we recognize that patient-specific parcellations, parcellation-free, or adaptive parcellation approaches may be beneficial (41–44).

Although we typically found high node strength in the resection area in good outcome patients, several high strength nodes were spared by surgery. For example, patient 1022 had multiple high strength nodes even in the contralateral hemisphere (Figure 3B). Given that all brain networks (epileptogenic or otherwise) have a mixture of high and low strength nodes, we interpret that seizures are facilitated by high strength nodes, but that not all high strength nodes are necessarily pathological. The normalization of patient networks against those from controls allows for the identification of pathological “abnormal” nodes (27, 45). Future studies should investigate these relationships between node hubness and node abnormality.

Despite several studies reporting relationships between structural MRI properties and epilepsy duration (46–48), few have investigated this relationship with MEG data. In agreement with our study, Madhaven et al. (13) performed an analysis of MEG data acquired from 12 patients with focal epilepsy and also found a significant positive correlation between connectivity and duration. In contrast to our analysis approach, the authors of that study analyzed only the subnetwork implicated in interictal epileptiform discharges, rather than performing a whole brain analysis as is presented here. Furthermore, that study reported significant findings only in beta band connectivity. However, our finding of increased mean network functional connectivity with increased epilepsy duration did not concur with Englot et al. (10). This may be due to the small size of the effect in our data (*R*^2^ < 0.2 for all segments), or the data used by Englot et al. (10) (*R*^2^ = 0.229). Other differences of note between the studies include the pre-processing strategies, and network types; specifically, Englot et al. (10) used alpha-band imaginary coherence, whereas our study uses broadband correlation. Given the limited and mixed literature, we conclude that a larger cohort with consistent processing is required to better understand relationships between duration and MEG functional connectivity.

An important limitation of this study is that the networks studied include neocortical areas only, and not deep brain structures including the amygdala or hippocampus. Previous work has demonstrated that MEG signals can be localized in deep brain structures. Pizzo et al. (49) showed that MEG signals could be source localized to spikes detected on concurrently recorded intracranial EEG. However, localization to the hippocampus is challenging (50). Given that our networks are constructed from low amplitude interictal activity, and that our objective was not high amplitude spike localization, we excluded those structures in our analysis. Other limitations of our study include the sample size used, which is in a similar range to previous studies (9), and the retrospective (as opposed to prospective) design of our analysis.

Prospective applications of our approach could involve using a mask of the intended resection overlaid with the patient's network as performed here. Calculation of an expected D_RS_, for the intended resection, could be made and this information used to alter the resection strategy. Multiple strategies could be computed and optimized for minimal D_RS_, minimal resection size, and maximal distance to eloquent areas. We envisage such a software tool could be used during pre-surgical evaluation (22).

This study has focussed on functional (MEG derived) network properties. Previous studies suggest that univariate properties (e.g., dominant frequency, inter-ictal spike rate, presence of HFOs) may also hold informative information (51–55). Although interictal spikes may be randomly present in any chosen epoch, their influence on functional connectivity is limited if they are present in only a minority of time windows. This influence is limited because our approach captures stationary aspects of the reconstructed network rather than the transient spikes (40, 56). However, we acknowledge that in circumstances where the majority of the recording contains spikes their influence on functional connectivity may be stronger. As we do not investigate spike counts here, this should be considered as a possible limitation of our work. Our approach as presented can be fully automated, without the need for manual identification of spikes, thus serving as a distinct advantage of our methods. Additional to neurophysiologically-derived networks, structural network information, which underpins functional network dynamics, has also been shown to have predictive value (22, 57, 58). Future studies should integrate univariate properties (such as spikes) with structural, and functional networks in a personalized patient specific manner to better understand the role of abnormal network hubs, maximizing the benefits of all patient data (59–61). Additionally, future studies should investigate robustness' of these results with different connectivity measures such as imaginary coherence or phase locked value (PLV).

Taken together, our study has provided additional evidence that the removal of network hubs can lead to improved patient outcomes from epilepsy surgery and suggested that these findings are temporally robust.

## Data Availability Statement

Preprocessed networks are available on reasonable request to the corresponding author. Requests to access the datasets should be directed to peter.taylor@newcastle.ac.uk.

## Ethics Statement

The studies involving human participants were reviewed and approved by Newcastle University Research Office Ref: 1804/2020. Written informed consent for participation was not required for this study in accordance with the national legislation and the institutional requirements.

## Author Contributions

SR, YW, NS, and PT conceived the presented idea. SR and PT performed the computations. AMc and AMi performed the surgery. JT organized data. FR-G and JD oversaw data collection. All authors provided input into the writing.

## Conflict of Interest

The authors declare that the research was conducted in the absence of any commercial or financial relationships that could be construed as a potential conflict of interest.
