# Supplementary material for: Removal of Interictal MEG-Derived Network Hubs Is Associated With Postoperative Seizure Freedom
Source: Front Neurol. 2020 Sep 24;11:563847. doi: 10.3389/fneur.2020.563847 (PMC7543719; doi:10.3389/fneur.2020.563847)
Supplement: Supplementary file 1 [file Table_1.pdf]

Supplementary material: Removal of interictal MEG-derived network hubs is associated with postoperative seizure freedom

Sriharsha Ramaraju[1], Yujiang Wang[1,2,3], Nishant Sinha[1,3], Andrew W McEvoy[2], Anna Miserocchi[2], Jane de Tisi[2], John S Duncan[2], Fergus Rugg-Gunn[2], Peter N Taylor[1,2,3*]

1. CNNP Lab (www.cnnp-lab.com), Interdisciplinary Computing and Complex BioSystems Group, School of Computing, Newcastle Helix, Newcastle University, UK
2. Department of Clinical and Experimental Epilepsy, UCL Queen Square Institute of Neurology, Queen Square, London WC1N 3BG, UK
3. Faculty of Medical Science, Newcastle University, UK

Supplementary Material

Table S1: Patient information. For binary variables 1 = true. Abbreviations: FCD; focal cortical dysplasia, HS; hippocampal sclerosis, EFS; end-folium sclerosis. N/A; data not available.

Patient ID	D_{RS} segment 1	D_{RS} segment 2	D_{RS} segment 3	Epilepsy duration	Outcome good	Type	Side	Pathology	Is female	Surgery age
'998'	0.216	0.288	0.228	17	0	T Lx	L	OTHER	0	31
'965'	0.660	0.582	0.523	20.2	0	P Lesx	R	FCD	0	32
'940'	0.277	0.409	0.410	5.7	0	T Lx	R	OTHER	0	25
'934'	0.510	0.410	0.529	10	1	O P Lx	R	OTHER	1	29
'931'	0.196	0.274	0.149	10	0	T Lx	L	HS (EFS)	0	30
'910'	0.673	0.683	0.593	12.3	0	F Lx	L	FCD	0	23
'874'	0.741	0.761	0.747	31	0	F Lx	L	FCD	1	38
'851'	0.440	0.760	0.701	24	0	F Lx	L	OTHER	1	43
'815'	0.333	0.150	0.365	9	0	T Lx	L	OTHER	1	24
'805'	0.234	0.206	0.478	8	0	T Lx	L	OTHER	1	32
'803'	0.355	0.311	0.382	18.5	0	T Lx	R	NAD	0	34
'782'	0.218	0.178	0.414	22	1	T Lx	L	OTHER	0	29
'1302'	0.583	0.497	0.432	41.1	1	T Lx	R	OTHER	0	47
'1284'	0.761	0.574	0.592	16.4	0	F Lx	R	FCD	0	21
'1236'	0.216	N/A	N/A	9.6	1	T Lx	R	HS	0	21
'1220'	0.494	0.463	0.341	11.6	0	T Lx	L	OTHER	0	27
'1216'	0.575	0.628	0.666	21.3	0	O P Lx	L	FCD	0	22
'1211'	0.267	0.317	0.483	13.2	0	T Lx	R	OTHER	0	26
'1210'	0.546	0.484	0.451	52.89	1	T Lx	L	HS	0	53
'1200'	0.264	0.471	0.244	24.34	1	T Lx	R	HS	1	25
'1182'	0.315	0.478	0.581	21.4	0	P Lesx	R	FCD	0	28
'1178'	0.742	N/A	N/A	15.3	0	F Lx	L	OTHER	1	23
'1168'	0.590	N/A	N/A	35.3	0	F Lx	L	FCD	1	60
'1163'	0.522	0.526	0.570	24.6	1	F Lx	L	FCD	1	28
'1142'	0.160	0.292	0.269	5.1	1	T Lx	R	OTHER	1	27
'1106'	0.579	0.630	0.713	21.5	0	F Lx	R	FCD	0	27
'1085'	0.156	0.193	0.253	13.9	1	F Lx	L	FCD	0	28
'1068'	0.125	0.102	0.340	26	1	T Lx	R	HS	1	48
'1063'	0.218	0.247	0.103	17	1	T Lx	R	OTHER	0	35
'1055'	0.282	0.304	0.289	33.7	0	T Lx	R	OTHER	0	48
'1022'	0.073	0.167	0.111	31.9	1	T Lx	R	OTHER	1	42

Table S2: Confusion matrices for DRS measure at threshold optimal for maximum sensitivity and specificity.

		Expected outcome	
Actual outcome	Segment 1	ILAE1	ILAE2-5
	ILAE1	16	3
	ILAE2-5	4	8

		Expected outcome	
Actual outcome	Segment 2	ILAE1	ILAE2-5
	ILAE1	15	2
	ILAE2-5	6	5

		Expected outcome	
Actual outcome	Segment 3	ILAE1	ILAE2-5
	ILAE1	14	3
	ILAE2-5	5	6

Table S3: AUC and P values for all parcellations and segments.

	**Num patients**	**68 ROIs**		**114 ROIs**		**219 ROIs**		**448 ROIs**	
		AUC	P val	AUC	P val	AUC	P val	AUC	P val
Segment 1	31	0.61	0.16	0.76	0.01	0.75	0.01	0.73	0.02
Segment 2	28	0.6	0.19	0.7	0.04	0.65	0.09	0.61	0.17
Segment 3	28	0.68	0.05	0.74	0.02	0.68	0.06	0.61	0.16

Table S4: Association between mean functional connectivity and duration of epilepsy

	**Num patients**	**R^2**	**P value**
Segment 1	31	0.098	0.030
Segment 2	28	0.002	0.098
Segment 3	28	0.170	<0.001

Table S5: Region volume and overlap information. (included as a separate attachment Microsoft excel file)
